# Healthcare Management and Quality during the First COVID-19 Wave in a Sample of Spanish Healthcare Professionals

**DOI:** 10.3390/nursrep11030051

**Published:** 2021-07-13

**Authors:** Patricia Torrent-Ramos, Víctor M. González-Chordá, Desirée Mena-Tudela, Laura Andreu Pejó, Celia Roig-Marti, María Jesús Valero-Chillerón, Águeda Cervera-Gasch

**Affiliations:** 1 Preventive Medicine Service, Hospital General de Castellón, 12071 Castellón, Spain; ptorrent@uji.es; 2 Nursing Department, Universitat Jaume I, 12071 Castellón, Spain; dmena@uji.es (D.M.-T.); pejo@uji.es (L.A.P.); chillero@uji.es (M.J.V.-C.); cerveraa@uji.es (Á.C.-G.); 3 Internal Medicine Service, Hospital General de Castellón, 12071 Castellón, Spain; celia.roig.marti@gmail.com

**Keywords:** COVID-19 pandemic, coronavirus, nursing, medical staff, healthcare quality, human resources, primary care, hospitals, nursing home

## Abstract

The aim of this study was to assess how the healthcare professionals in the Castellón Province (Spain) perceive healthcare quality and management during the first COVID-19 wave. A cross-sectional study was carried out. An online survey on healthcare quality and management during the first COVID-19 wave was sent to healthcare professionals. Almost half of the sample believed that healthcare quality worsened during the first COVID-19 wave (45.3%; *n* = 173). Heavier workload (m = 4.08 ± 1.011) and patients’ complexity (m = 3.77 ± 1.086) were the factors that most negatively impacted healthcare quality. Health department 3, primary care center, and other doctors assessed human and material resources management as significantly worse (*p* < 0.05). Human and material resources management and the healthcare organization negatively affected healthcare quality during the first COVID-19 wave. Significant differences were observed according to departments, services, and professionals.

## 1. Introduction

Spain has one of the best health systems in the world [1] and occupies position 15 in the Global Health Security Index ranking [2]. Nevertheless, data indicate Spain as one of the countries to be most affected by the COVID-19 pandemic, and some experts stress the need to individually evaluate how Spain has responded to this pandemic [3]. While waiting for this evaluation to be made, healthcare professionals lived the consequences of the taken measures first-hand and have witnessed the possible impact on healthcare quality.

COVID-19 is an infectious disease caused by a new kind of coronavirus known as SARS-CoV-2 [4]. This virus is transmitted via direct contact or when an infected person releases droplet while talking, coughing or sneezing [5], and possibly via aerosols [6]. Although some cases are asymptomatic, the virus is initially manifested by mild respiratory symptoms after 4–8 incubation days, and can become clinically serious with pneumonia, multisystem failure, and even death, which occur mainly in people with previous diseases [7].

The first cases of COVID-19 disease were detected in the Hubei Province (China) at the end of 2019. The new coronavirus rapidly spread to other Asian countries and had reached Europe by the end of January 2020. The World Health Organization (WHO) declared a pandemic by SARS-CoV-2 on 11 March 2020, with 118,000 cases in 114 countries [8]. There were more than 152 million infected people and almost 3 million worldwide on 1 May 2021 [9].

SARS-CoV-2 has a limited capacity to produce serious disease and its mortality is estimated at 4.8% (95% CI: 1.00–11.4) [7]. However, its marked capacity to transmit this virus and the rapid growing number of cases in a short time led to an unforeseeable increase in the demand and requirement of infrastructures, as well as human and material resources. This meant that healthcare systems all over the world came to a standstill, which compromised healthcare quality [10]. 

In Spain, the first imported COVID-19 case was notified on 30 January 2020. The increasing number of COVID-19 cases led the Spanish Government to declare a state of alarm that lasted 3 months and 7 days, from 14 March to 21 June [11]. The state of alarm is a legislative instrument contemplated by the Spanish Constitution, which temporarily concentrates power in governments and allows them to make unilateral decisions. This measure can be taken in exceptional situations, such as natural catastrophes or healthcare crises [12].

The intention of this state of alarm was to stop the virus from spreading and to flatten the curve of contagions [13]. To do so, and according to how the curve of contagions progressed, different physic-social distancing measures were taken while the state of alarm lasted, such as restricting the population’s movements to shop and purchase medicines, closing public spaces, wearing masks, confining the population, and not performing any non-essential occupational activity during a 15-day period.

Apart from taking these measures to prevent the virus from spreading, other measures were taken to avoid blocking health services, and to ensure that infrastructures and human and material resources were available [14]. Another approved measure was for public health services to manage private health services. The Spanish Government also centralized purchases of the material resources and personal protective equipment (PPE) needed to prevent professionals from catching the virus while attending COVID-19 patients. Retired healthcare professionals were also authorized to return to work, and final-year nursing and medicine students were contracted to work [15]. 

All these measures have implied relevant changes in the organization of health services and, specifically, in nursing services. Despite the limited literature available so far on how nursing managers and registered nurses are dealing with the organization of health services to cope with the pandemic, recent studies in Spain show the magnitude of decisions and the speed with which they are being taken. This is due to the overwhelming need to increase the nursing workforce, reorganize the organizational model of care, and ensure the availability of material resources [16,17].

Despite all these measures, accumulated cases went from 4231 to 246,835 in 3 months [18], which pushed the operational capacity of Spanish health services to the limit. Recent studies informed how inpatient units were transformed into intensive care units [19] or how healthcare professionals caught the virus because they had no PPE [20]. Hence, the objective of this study was to assess how healthcare professionals from the Castellón Province (Spain) perceive healthcare quality and management during the first COVID-19 wave.

## 2. Materials and Methods

### 2.1. Design and Setting

A cross-sectional study with an online survey was conducted in the Castellón Province (E Spain), where the health system is organized into three health departments, each with a reference hospital and different primary care centers that each cover populations between 200,000 and 250,000 inhabitants. One of the health departments has two other hospitals: one specializes in oncology and mental health, while the other is used for chronic patients and rehabilitation. This province has 40 nursing homes for the elderly and disabled. Of these, 60% (*n* = 24) are private homes and the rest are public. Private healthcare is limited to one hospital, with rooms for medical specialists and hemodialysis clinics.

### 2.2. Participants and Sample

Our study population included the healthcare professionals who worked in the various services offered in the Castellón Province in both private and public healthcare. According to the most recently available data, in 2019 there were 2959 registered nurses and 2667 medical practitioners (doctors). No data are available about other groups, such as nursing assistants, and data do not differ between public and private systems or between the different types of center and service [21]

### 2.3. Variables

An online survey devised with Google Docs was forwarded. It included 28 questions arranged into different blocks. The first block of seven questions asked the professionals if they thought that healthcare quality had worsened, remained the same, or had improved during the state of alarm. They were also asked to assess the impact of different factors on healthcare quality (workload, human resources, material resources, teamwork, patients’ clinical complexity, and healthcare organization). These questions were answered on a 5-point Likert-type scale (1: Strong negative impact; 5: Strong positive impact). 

The second block contained 11 questions about managing human and material resources. The professionals were asked to assess on an ascending 5-point Likert-type scale (1: Not at all appropriate; 5: Most appropriate) the staff reinforcement contracts signed and contract duration. They were also asked to assess on an ascending 5-point Likert-type scale the availability of different material resources (surgical gloves, protective face shields, impermeable gowns, cleaning and disinfecting products, and other material resources). Two questions were included about the training received in handling PPE and cleaning/disinfecting products.

The third block comprised 10 questions about how health care was organized during the state of alarm. They were asked to specifically assess on an ascending 5-point Likert-type scale (1: Not very appropriate; 5: Most appropriate) how centers’ management responded, supervisors’ direct concern about work teams’ well-being, how work was organized, the clarity of protocols, and the suitability of the circuits set up to attend to COVID-19 patients. Two questions were also included about the training received in the new organization of both work and teamwork. The professionals were also requested to assess if their occupational rights and conditions were respected during the state of alarm. Finally, they were asked to assess if the health system was ready to face a new outbreak.

We collected socio-demographic variables: age, gender, and family responsibilities (children, elderly people, dependent people), as well as perceived health status (very good, good, normal, bad). Occupational variables were also included, such as type of center (public; private), the health department they belonged to (HD1; HD2; HD3), healthcare service (primary care center; hospital; nursing home; others (healthcare transport or private offices, among others)), their professional category (doctor; nurse; nursing assistant; others (hospital porter or technicians, among other)), their contract type (temporary; permanent; contracted specially for the pandemic; substitution; resident in training; other), years of experience (less than 5; between 5 and 10; between 10 and 15; more than 15), and other variable related to COVID-19 exposure (positive case, diagnosis technique, isolation).

### 2.4. Data Collection

Data collection took place between 1 and 15 July 2020. The online survey was diffused on social networks like Facebook, Twitter, Instagram, or WhatsApp as they are the most widely used in Spain. The recommendations by Pedersen and Kurz [22] about using social networks for data collection were followed.

### 2.5. Data Analysis

A descriptive analysis was carried out of the variables included in this study in line with their nature. The comparison of the results according to health service, health department, and professional category was made using the Kruskal–Wallis H test after confirming that groups did not follow normal distribution. The categories nursing homes and other services were grouped for poor representativeness. A chi-squared test (X2) was used with the qualitative variables. It was not necessary to address missing data. The statistical analysis was done by the SPSS V21 software (IBM, New York, EEUU). The level of significance was set at *p* < 0.05.

### 2.6. Ethical Considerations

This study was designed in accordance with Spanish Organic Law 03/2018 on Personal Data Protection and Guaranteeing Digital Rights. Additional provision 17 on processing of health data, paragraph 2g, specifies that studies with pseudomized or anonymized data must be submitted for evaluation by an ethics and research committee. This study did not experiment with human beings and the participants answered voluntarily and anonymously and no personal data that could identify each participant, email, or IP address were collected to guarantee confidentiality, so it did not need approval by an ethics and research committee. The first page of the survey included information on the study objective and methodology, along with a box with which the participants gave their informed consent, confirming that their participation was voluntary and anonymous. Moreover, Declaration of Helsinki Principles were respected (charity, non-maleficence, autonomy, justice).

## 3. Results

### 3.1. Sample Characteristics

We collected 382 surveys. The participants’ mean age was 42.97 (95% CI: 41.94–44.00) years; 82.2% (*n* = 314) were female and 53.7% (*n* = 205) considered that their health status was good; 63.4% (*n* = 242) had children, and 21.7% (*n* = 83) looked after elderly people. Most of the participants in our sample were registered nurses (56.8%; *n* = 217); 54.7% (*n* = 209) of the sample had more than 15 years of experience and 52.4% (*n* = 209) had temporary contracts. Most of the participants belonged to the public health system (96.9%; *n* = 370), specifically from HD2 (74.6%; *n* = 285), they worked in a hospital (74.6%; *n* = 285), and 84.8% (*n* = 324) had been in contact with COVID-19 patients. Only 15.7% (*n* = 60) stated that they had had a diagnosis test and 18.3% (*n* = 70) had been isolated. The prevalence of COVID-19 cases in the study sample was 7.6% (*n* = 29). Table 1 shows the analysis of the socio-demographic, occupational, and COVID-19 exposure-related variables.

### 3.2. Healthcare Quality

Healthcare quality was perceived by 45.3% (*n* = 173) of our sample as becoming worse during the first COVID-19 wave, while the other respondents stated that the same conditions remained (43.7%; *n* = 167) or improved (11%; *n* = 42). No significant differences were found for health department (H = 4.007; *p* = 0.405) or service type (H = 7.355; *p* = 0.499). Nevertheless, significant differences appeared according to professional category because doctors (60.9%; *n* = 42) and registered nurses (44.7%; *n* = 97) were the professional groups that mainly thought that healthcare quality worsened during the state of alarm (H = 14.36; *p* = 0.026). 

Workload was assessed as the factor with the most negative impact on healthcare quality (m = 1.92 ± 1.011) (H = 4.189; *p* = 0.123), followed by patients’ clinical complexity (m = 2.23 ± 1.086) (H = 0.02; *p* = 0.99), but no significant differences were found according to healthcare service. Another factor with a negative impact on healthcare quality was material resources management (m = 2.35 ± 1.228), mostly in primary care centers (m = 1.88 ± 1.045) as opposed to nursing homes and other services (m = 2.29 ± 1.243) and hospitals (m = 2.46 ± 1.243), with significant differences (H = 12.616; *p* = 0.002). 

Human resources management was assessed at 2.38 (±1.180) points. The professionals who worked in primary care centers (m = 2.11 ± 1.069), as well as nursing homes and other services (m = 2.1 ± 1.012), indicated that this factor had the stronger negative impact versus those professionals who worked in hospitals (m = 2.48 ± 1.209), with significant differences (H = 6.655; *p* = 0.036). Health service organizations obtained a score of 2.77 (±1.278) points, and no significant differences were found according to health service (H = 0.65; *p* = 0.723).

Finally, teamwork was considered to be the only factor with a positive impact on healthcare quality (m = 3.60 ± 1.255), mainly in hospitals (m = 3.70 ± 1.255) vs. primary care centers (m = 3.26 ± 1.281) and nursing homes and other services (m = 3.35 ± 1.05), with significant differences (H = 0.65; *p* = 0.011). Table 2 offers these results for health department and professional category.

### 3.3. Managing Human and Material Resources

In general terms, human resources management during the state of alarm obtained 3.07 (±1.210) points and no significant differences appeared for health services (H = 3.053; *p* = 0.217). The number of staff reinforcement contracts obtained 3.16 (±1.343) points, with a worse score for primary care centers (m = 2.42 ± 1.29) and nursing homes and other services (m = 2.97 ± 1.449) than for hospitals (m = 3.35 ± 1.285), with significant differences (H = 25.231; *p* < 0.001). The time that reinforcement staff contracts lasted (2.95 ± 1.379) was scored worse in primary care centers (m = 2.36 ± 1.211) and nursing homes and other services (m = 2.45 ± 1.457) than in hospitals (m = 3.14 ± 1.359), with significant differences (H = 21.422; *p* < 0.001).

Availability of protective face shields scored 2.88 (±1.446) points, and the score given by the professionals for working in hospitals was statistically higher (m = 3.06 ± 1.438) than in primary care centers (m = 2.36 ± 1.32) and nursing homes and other services (m = 2.42 ± 1.455) (H = 15.956; *p* < 0.001). The availability of impermeable gowns (m = 2.91 ± 1.358) and masks (m = 2.94 ± 1.336) obtained a significantly higher score in hospitals (gowns m = 3.12 ± 1.332; masks m = 3.11 ± 1.31) than in primary care centers (gowns m = 2.24 ± 1.151; masks m = 2.41 ± 1.24) and in nursing homes and other services (gowns m = 2.45 ± 1.457; masks m = 2.48 ± 1.411) (gowns H = 26.277, *p* < 0.001; masks H = 18.209, *p* < 0.001). Similar results were found for the availability of cleaning and disinfecting materials (m = 3.65 ± 1.234), which were significantly better assessed in hospitals (m = 3.76 ± 1.211) (H = 9.899; *p* = 0.007).

Finally, no significant differences were observed for healthcare professionals’ assessments according to the health service about the availability of other material resources required to attend to patients (m = 3.26 ± 1.148) (H = 2.801; *p* = 0.247) and the availability of surgical gloves (m = 3.6 ± 1.276) (H = 0.38; *p* = 0.827). Nor were significant differences observed for training in handling PPE (m = 2.80 ± 1.294) (H = 1.747; *p* = 0.418) and training in the use of cleaning and disinfecting products (m = 2.84 ± 1.282) (H = 2.972; *p* = 0.226). Table 3 shows the results of human/material resources management according to health department and professional category.

### 3.4. Healthcare Organisation

The whole sample gave 3.36 (±1.147) points for the response of healthcare centers’ management to the COVID-19 pandemic. The professionals who worked in primary care gave a significantly better score (m = 3.61 ± 1.456) than those who worked in hospitals (m = 3.21 ± 1.169) and in nursing homes and other services (m = 2.97 ± 1.169) (H = 10.552; *p* = 0.005). Supervisors’ concern for work teams’ well-being was scored 3.74 (±1.316) points, and there were no significant differences for health services (H = 2.862; *p* = 0.239).

How work was organized obtained a mean score of 3.57 (±1.175) points and was significantly better assessed in primary care centers (m = 3.89 ± 1.125) than in hospitals (m = 3.53 ± 1.179) and nursing homes and other services (m = 3.26 ± 1.125) (H = 8.747; *p* = 0.013). No significant differences appeared in the assessments of either the healthcare protocols set up (m = 2.70 ± 1.281) (H = 1.853; *p* = 0.396) or the suitability of the circuits set up to attend to COVID-19 patients (m = 3.20 ± 1.091; *p* = 0.65).

No significant differences were found in the assessments of either training received (m = 2.71 ± 1.203) (H = 1.824; *p* = 0.402) or teamwork (m = 4.13 ± 1.115) (H = 3.418; *p* = 0.402) according to healthcare service. However, primary care professionals (m = 2.59 ± 1.425) assessed respect for their occupational rights as being significantly worse than those professionals from hospitals (m = 3.06 ± 1.336) and nursing homes and other services (m = 2.74 ± 1.483) (H = 6.661; *p* = 0.36); primary care professionals (m = 2.61 ± 1.402) assessed their working conditions as being significantly worse than those from nursing homes and other services (m = 2.9 ± 1.446) and hospitals (m = 3.04 ± 1.365) (H = 3.04 ± 1.365).

Finally, preparing health services to face a new COVID-19 outbreak obtained a score of 2.99 (±1.234) points, and significant differences were found depending on the service that the professionals worked for (H = 6.262; *p* = 0.027). Primary care professionals gave a lower score (m = 2.68 ± 1.23), followed by the professionals from hospitals (m = 3.02 ± 1.217), and finally nursing homes and other services (m = 3.32 ± 1.301). Table 4 shows the analysis done of these matters according to health department and professional category.

## 4. Discussion

A well-organized and prepared health system should have the capacity to maintain reasonable access to high-quality health services during a healthcare emergency. This capacity depends on a coordinated response from health authorities, having contingency plans that allow for health services to be organized, clear protocols to attend to patients, and suitable human and material resources management [23]. Today the COVID-19 pandemic challenges the operation and sustainability of health systems worldwide, with differences among countries as far as measures taken and the obtained results [24]. Initially in Spain, a virus containment model was adopted and the national government centralized decision-making [13]. However, subsequent decisions seem to have been taken from a perspective of living with the virus and decision-making returned to the regional governments. It is convenient to remember that Spain is a decentralized country where health competences, among others, are transferred to regional governments. The measures adopted by the central government have an impact at the regional level and this can be observed in the interregional differences in the evolution of the pandemic [25], although the magnitude of this impact must be confirmed in future studies.

Nearly 50% of the healthcare professionals who participated in this study believed that healthcare quality worsened during the first COVID-19 wave. Doctors and registered nurses were the groups that assessed healthcare quality as worse during this period, probably because they were the professionals who worked in the first healthcare line of attention, and who endured a very heavy physical, psychological, and social load [26]. Indeed, more than 80% stated having been in contact with COVID-19 patients, although only 15.7% of the surveyed professionals had diagnostic tests. The prevalence of the healthcare professionals with COVID-19 in Spain was 20% but was 7.6% in this study. Nonetheless, the quantity of infected professionals varied from one region of Spain to another, and official data for provinces are not available to compare these results [27].

Workload was assessed as the factor that most impacted healthcare quality. In fact, a recent study indicates how the nursing workload was heavier when working with COVID-19 patients than with non-COVID-19 patients in an intensive care unit [28]. Nevertheless, it is striking that registered nurses were not the professional group that assessed human resources management, hiring staff, or respecting occupational rights as worse, according to the large body of evidence for occupational precariousness and shortage of registered nurses in Spain, with a nurse-patients ratio below the mean reported by the OECD [29]. Despite the current situation of the nursing workforce in Spain, which has been tremendously complicated by the pandemic, it is very possible that the humanistic values of the profession, its willingness to serve people who need it, and its capacity of resilience can explain these results, coinciding with other studies [20,30].

The differences encountered in health departments on the impact that workload had on healthcare quality, as well as other aspects on human and material resources management and organizing health care, can be explained by high healthcare pressure due to COVID-19 cases on HD2 and HD3 compared to HD1 [31]. Nevertheless, benchmarking techniques will help to detect the possible differences in the strategies adopted in the three health departments [32].

Moreover, those professionals who worked in primary care, nursing homes, and other services assessed human and material resources management worse. Spain’s initial response came late and primary care strategies were not developed to contain SARS-CoV-2 from spreading, which coincided with the seasonal flu epidemic [33]. Moreover, the pandemic evidenced the precarious situation of nursing homes in Spain [34]. Therefore, all efforts had to center on supplying hospitals in order to attend to the increasing general number of cases and serious cases. 

The healthcare organization assessment can be considered appropriate. Nonetheless, the primary care professionals better assessed their organization than that in hospitals, nursing homes, and other services. As previously mentioned, hospitals received most of the patients with this new disease caused by the virus that had recently appeared. Modes of viral transmission, its risk factors, clinical evolution, symptoms, or treatments for this disease are being investigated as the pandemic advances. These factors could have had an influence when setting up suitable circuits and clear protocols to attend to these patients, which could have made healthcare organization difficult. Another point to stress is the poor assessment that the professionals made of previous training in using PPE, disinfection, cleaning, and the new work organization. The WHO considers that training and supporting professionals are fundamental in this healthcare emergency [24].

The results of this work must be taken cautiously. On the one hand, this study was conducted only about the healthcare professionals working in one province in Spain and the impact of the first COVID-19 wave was variable. Professionals from other Spanish regions may have different views about healthcare management and quality. Even the opinion and perception of the same group of professionals may vary depending on their field of work. For example, there could be differences between the perception of registered nurses who work in hospitals, health centers, or nursing homes. However, our sample was limited and not randomized, which prevents some variables from being compared, e.g., if services were public or private. These types of analyses should be addressed in future studies, with representative and larger samples. In addition, there are not enough data available to determine the representativeness of the sample on the population studied according to the type of health system, department, center, or service. Another important aspect is related to the data collection instrument, since a survey was used instead of a validated questionnaire. This can affect the reliability of the results.

Other studies carried out in Spain, with online surveys and similar limitations, focused on studying the quality of life of healthcare professionals during the first COVID-19 wave [35] or the factors related to SARS-CoV-2 infection in healthcare professionals [36]. However, despite these limitations, our results are interesting because no previous studies about how Spanish healthcare professionals assess healthcare management and quality during an epidemic outbreak were found, possibly because Spain has not recently been affected by serious epidemic outbreaks. Knowing how healthcare professionals assess healthcare quality and management during the first COVID-19 wave is important. The outcomes of this study can help to detect aspects that can improve when preparing the health system for a new wave of COVID-19 or other infectious diseases. Specifically, it was observed how the emergency situation caused by the COVID-19 pandemic increased the needs of the workforce and material resources, in addition to requiring a new organization of patient care. Decision makers and managers of health services should seriously consider these factors when preparing contingency plans.

## 5. Conclusions

Overall, 45% of the healthcare professionals from the Castellón Province (Spain) consider that healthcare quality worsened during the first COVID-19 wave. The factors that negatively impacted healthcare quality were heavier workload and patients’ complexity, both of which are related to human/material resources management and healthcare organization. Significant differences were observed according to health department, type of health service, and type of professional, and studies with bigger samples should deal with these variables in the future.

## Figures and Tables

**Table 1:** Socio-demographic, occupational, and COVID-19 exposure-related variables.

Title 1	Title 2	%(*n*)
Gender	Man	17.3 (68)
	Woman	82.2 (314)
Have children	Yes	63.4 (242)
	No	36.6 (140)
Look after elderly people	Yes	21.7 (83)
	No	78.3 (299)
Look after dependent people	Yes	11.5 (44)
	No	88.5 (338)
Health status	Very good	34.8 (133)
	Good	53.7 (205)
	Normal	11 (42)
	Bad	0.5 (2)
Health department	HD1	5.2 (20)
	HD2	74.6 (285)
	HD3	20.2 (77)
Type of center	Public	96.9 (370)
	Private	2.9 (11)
Professional category	Doctors	18.1 (69)
	Registered nurses	56.8 (217)
	Nursing assistants	18.1 (69)
	Others (hospital porters, technicians)	7.1 (27)
Years of experience	<5	12.6 (48)
	5–10	13.6 (52)
	10–15	19.1 (73)
	>15	54.7 (209)
Type of contract	Temporary Permanent Reinforcement for pandemic Substitution Resident in training Other	52.4 (200) 27.2 (104) 8.4 (32) 6.0 (23) 4.5 (4.5) 1.6 (6)
Service	Health center	17.3 (66)
	Hospital	74.6 (285)
	Nursing home and others	8.1 (31)
Contact with COVID-19 patients	Yes	84.8 (324)
	No	15.2 (58)
Positive COVID-19	Yes	7.6 (29)
	No	89.5 (342)
	I’d rather not answer	2.9 (11)
Isolation	Yes	18.3 (70)
	No	80.9 (309)

**Table 2:** Factors that negatively affected healthcare quality according to health department and professional category.

Factors	Health Department (m; sd)				Professional Category (m; sd)				
	HD1	HD2	HD3	*p*	Nurses	Doctors	Assistants	Others	*p* ^1^
Workload	2.25 (1.209)	1.97 (1.026)	1.64 (0.842)	0.017	1.98 (1.056)	1.67 (0.918)	1.90 (0.91)	2.11 (1.05)	0.078
Human resources	2.75 (1.517)	2.40 (1.181)	2.25 (1.066)	0.383	2.60 (1.229)	1.84 (1.038)	2.23 (1.002)	2.44 (1.086)	<0.001
Material resources	2.65 (1.387)	2.39 (1.242)	2.10 (1.107)	0.135	2.53 (1.277)	1.91 (1.011)	2.26 (1.221)	2.22 (1.086)	0.005
Teamwork	3.70 (1.174)	3.53 (1.263)	3.83 (1.229)	0.134	3.73 (1.21)	3.48 (1.313)	3.43 (1.377)	3.22 (1.013)	0.065
Clinical complexity	2.55 (1.05)	2.23 (1.082)	2.14 (1.109)	0.261	2.22 (1.051)	2.26 (1.066)	2.22 (1.223)	2.30 (1.103)	0.950
Healthcare organization	3.00 (1.622)	2.75 (1.224)	2.77 (1.385)	0.828	2.69 (1.266)	2.97 (1.35)	2.83 (1.224)	2.70 (1.325)	0.468

^1^ Level of significance was set at *p* < 0.05.

**Table 3:** Results of human/material resources management according to health department and professional category.

Questions	Health Department (m; sd)				Professional Category (m; sd)				
	HD1	HD2	HD3	*p*	Nurses	Doctors	Assistants	Others	*p* ^1^
Human resources management	3.65 (1.268)	3.04 (1.204)	3 (1.192)	0.102	3.2 (1.172)	2.68 (1.169)	2.99 (1.3)	3.19 (1.21)	0.023
Reinforcement contracts	3.7 (1.525)	3.13 (1.319)	3.13 (1.370)	0.143	3.44 (1.228)	2.04 (1.130)	3.46 (1.313)	2.96 (1.344)	<0.001
Contract duration	3.65 (1.755)	2.94 (1.339)	1.79 (1.380)	0.51	3.17 (1.389)	2.07 (1.048)	3.2 (1.279)	2.78 (1.423)	<0.001
Availability of masks	3.20 (1.399)	3.02 (1.317)	2.55 (1.333)	0.015	3.16 (1.306)	2.68 (1.312)	2.48 (1.335)	3 (1.301)	0.001
Availability of surgical gloves	3.9 (1.294)	3.58 (1.263)	3.57 (1.322)	0.468	3.84 (1.177)	3.32 (1.388)	3.12 (1.334)	3.59 (1.152)	<0.001
Availability of gowns	3.1 (1.334)	3.06 (1.318)	2.34 (1.373)	0.001	3.12 (1.329)	2.49 (1.324)	2.70 (1.354)	2.85 (1.406)	0.003
Availability of face shields	3.6 (1.603)	2.88 (1.428)	2.70 (1.433)	0.053	3.16 (1.465)	2.38 (1.373)	2.57 (1.323)	2.78 (1.311)	<0.001
Availability of other material resources	3.5 (1.235)	3.28 (1.138)	3.13 (1.162)	0.317	3.44 (1.104)	2.83 (1.2)	3.19 (1.154)	3.19 (1.075)	0.001
Availability of cleaning products	4.05 (1.317)	3.69 (1.191)	3.42 (1.341)	0.034	3.85 (1.161)	3.2 (1.208)	3.64 (1.317)	3.26 (1.318)	0.004
Training in PPE	3.4 (1.667)	2.83 (1.284)	2.53 (1.165)	0.063	2.93 (1.345)	2.51 (1.066)	2.94 (1.282)	2.15 (1.167)	<0.001
Training in cleaning/disinfecting products	3.15 (1.663)	2.93 (1.213)	2.39 (1.339)	0.002	2.86 (1.030)	2.58 (1.218)	3.1 (1.274)	2.63 (1.214)	0.091

^1^ Level of significance was set at *p* < 0.05.

**Table 4:** Healthcare organization according to health department and professional category.

Questions	Health Department (m; sd)				Professional Category (m; sd)				
	HD1	HD2	HD3	*p*	Nurses	Doctors	Assistants	Others	*p* ^1^
Management’s response	3.8 (1.152)	3.19 (1.121)	3.38 (1.023)	0.034	3.19 (1.297)	3.61 (1.297)	3.07 (1.048)	3.37 (1.275)	0.006
Concern about work teams	4.45 (1.05)	3.68 (1.308)	3.75 (1.368)	0.02	3.79 (1.035)	3.84 (1.302)	3.61 (1.297)	3.33 (1.468)	0.267
How work was organized	3.95 (1.146)	3.49 (1.174)	3.78 (1.154)	0.036	3.59 (1.132)	3.87 (1.123)	3.33 (1.291)	3.3 (1.203)	0.025
Clarity of protocols	3.35 (1.348)	2.68 (1.213)	2.62 (1.170)	0.071	2.7 (1.258)	2.74 (1.159)	2.65 (1.161)	2.81 (1.241)	0.928
COVID-19 circuits	3.60 (1.046)	3.15 (1.098)	3.29 (1.062)	0.087	3.24 (1.092)	3.22 (0.998)	3.10 (1.113)	3.11 (1.281)	0.837
Training	3.35 (1.599)	2.67 (1.185)	2.68 (1.117)	0.019	2.66 (1.249)	2.86 (1.102)	2.72 (1.123)	2.67 (1.301)	0.585
Teamwork	4.35 (1.268)	4.06 (1.124)	4.32 (1.019)	0.046	4.23 (1.042)	4.12 (1.182)	4.01 (1.266)	3.67 (1)	0.027
Occupational rights	3.45 (1.504)	3.03 (1.350)	2.53 (1.334)	0.005	3.09 (1.322)	2.33 (1.268)	2.88 (1.44)	3.59 (1.338)	<0.001
Working conditions	3.20 (1.576)	3.02 (1.378)	2.66 (1.334)	0.1	3.13 (1.344)	2.10 (1.214)	3.14 (1.320)	3.26 (1.059)	<0.001
Ready for a new outbreak	2.95 (1.538)	2.98 (1.163)	3.04 (1.409)	0.886	3.02 (1.215)	2.64 (1.2)	3.09 (1.280)	3.37 (1.214)	0.039

^1^ Level of significance was set at *p* < 0.05.

## Data Availability

Data are available upon request to the authors.
